# A Multicenter Retrospective Survey regarding Diabetic Ketoacidosis Management in Italian Children with Type 1 Diabetes

**DOI:** 10.1155/2016/5719470

**Published:** 2015-11-15

**Authors:** Stefano Zucchini, Andrea E. Scaramuzza, Riccardo Bonfanti, Pietro Buono, Francesca Cardella, Vittoria Cauvin, Valentino Cherubini, Giovanni Chiari, Giuseppe d'Annunzio, Anna Paola Frongia, Dario Iafusco, Giulio Maltoni, Ippolita Patrizia Patera, Sonia Toni, Stefano Tumini, Ivana Rabbone, Diabetes Study Group of the Italian Society for Pediatric Endocrinology and Diabetology (ISPED)

**Affiliations:** ^1^Department of Pediatrics, S. Orsola-Malpighi Hospital, Via Albertoni 15, 40138 Bologna, Italy; ^2^Department of Pediatrics, Azienda Ospedaliera, “Ospedale Luigi Sacco”, University of Milan, Via G.B. Grassi 74, 20157 Milan, Italy; ^3^Department of Pediatrics, Endocrine Unit, Scientific Institute Hospital San Raffaele, Vita-Salute University, Via Olgettina 60, 20132 Milan, Italy; ^4^UOSD Pediatric Diabetology, ASL NA2 Nord, Via Corrado Alvaro 8, Monteruscello, 80072 Pozzuoli, Italy; ^5^Department of Pediatrics, U.O.S. Pediatric Diabetology, ARNAS Civico Di Cristina, Via Benedettini 1, 90134 Palermo, Italy; ^6^Pediatric Unit, S. Chiara Hospital, Largo Medaglie d'Oro 9, 38122 Trento, Italy; ^7^Regional Center for Diabetes in Children and Adolescents, Department of Woman and Child Health, AOU Salesi Hospital, Via Corridoni 11, 60123 Ancona, Italy; ^8^Postgraduate School of Pediatrics, University of Parma, Viale Gramsci 14, 43100 Parma, Italy; ^9^Clinica Pediatrica, IRCCS Istituto Giannina Gaslini, Via Gaslini 5, 16147 Genova, Italy; ^10^Unit of Pediatric Diabetes, Brotzu Hospital, Piazzale Ricchi 1, 09134 Cagliari, Italy; ^11^Department of Pediatrics, Second University of Naples, Via S. Andrea delle Dame 4, 80138 Naples, Italy; ^12^Endocrinology and Diabetes Unit, University Department of Pediatric Medicine, Bambino Gesù Children's Hospital, Piazza Sant'Onofrio 4, 00165 Rome, Italy; ^13^Juvenile Diabetes Center, Anna Meyer Children's Hospital, Via Pieraccini 24, 50132 Florence, Italy; ^14^Center of Pediatric Diabetology, University of Chieti, 66100 Chieti, Italy; ^15^Department of Pediatrics, University of Turin, Piazza Polonia 94, 10126 Turin, Italy

## Abstract

We conducted a retrospective survey in pediatric centers belonging to the Italian Society for Pediatric Diabetology and Endocrinology. The following data were collected for all new-onset diabetes patients aged 0–18 years: DKA (pH < 7.30), severe DKA (pH < 7.1), DKA in preschool children, DKA treatment according to ISPAD protocol, type of rehydrating solution used, bicarbonates use, and amount of insulin infused. Records (*n* = 2453) of children with newly diagnosed diabetes were collected from 68/77 centers (87%), 39 of which are tertiary referral centers, the majority of whom (*n* = 1536, 89.4%) were diagnosed in the tertiary referral centers. DKA was observed in 38.5% and severe DKA in 10.3%. Considering preschool children, DKA was observed in 72%, and severe DKA in 16.7%. Cerebral edema following DKA treatment was observed in 5 (0.5%). DKA treatment according to ISPAD guidelines was adopted in 68% of the centers. In the first 2 hours, rehydration was started with normal saline in all centers, but with different amount. Bicarbonate was quite never been used. Insulin was infused starting from third hour at the rate of 0.05–0.1 U/kg/h in 72% of centers. Despite prevention campaign, DKA is still observed in Italian children at onset, with significant variability in DKA treatment, underlying the need to share guidelines among centers.

## 1. Introduction

Diabetic ketoacidosis (DKA) is a metabolic derangement characterized by the triad of hyperglycemia, acidosis, and ketosis that occurs in the presence of very low levels of effective insulin action. DKA is a current threat for children with type 1 diabetes and its epidemiology is variable in various countries [1, 2]. The frequency of DKA at diagnosis ranges from 12.8% to 80% and is lowest in Sweden, the Slovak Republic, and Canada and highest in the United Arab Emirates, Saudi Arabia, and Romania [1]. The frequency of DKA is lower in countries where the background incidence of type 1 diabetes is higher and in countries further from the equator and with a lower gross domestic product [1].

All DKA episodes are theoretically preventable when care of children with type 1 is appropriate [3] and specific prevention campaigns are carried out [4]. However, to date, DKA is still a problem for patients with type 1 diabetes.

For this reason, several national and international guidelines on DKA treatment and management have been published [5–8], even if a real agreement (grade A evidence) is still lacking, except on a few key points.

Incidence and rate of complications of DKA and its treatment are largely dependent on various parameters including type 1 diabetes incidence in a certain area, age of the child, and access to care of patients and their families [9].

Epidemiology of DKA at diabetes diagnosis in Italy dates back to quite 10 years ago [10] and included only few areas with data coming from regional centers [11].

In Italy health care is provided free of charge by family pediatricians throughout the country and once diagnosed quite all children are managed by pediatricians belonging to the Italian Society for Pediatric Endocrinology and Diabetology (ISPED), many of whom work in tertiary referral centers.

The aim of the present study is to evaluate DKA incidence at diagnosis in Italy during the calendar years 2012-2013 in subjects 0–18 years old and to evaluate similarities or differences in DKA management among centers.

## 2. Methods

A survey to collect recent data and investigate DKA epidemiology and management has been given to all pediatricians belonging to the Diabetes Study Group of the ISPED (*n* = 77).

Each center referring to the ISPED has been asked to review all records of patients under 19 years of age, who had diabetes onset between January 1, 2012, and December 31, 2013.

For each patient several pieces of information have been requested as well as for the center itself: in detail for the patients, date of birth, date at diabetes onset, pH value at diabetes onset (to evaluate DKA severity), which kind of DKA treatment has been used (if any protocol has been followed, e.g., ISPAD protocol for DKA or others), measurement of beta-hydroxybutyrate in the blood (yes or no), and any treatment complication (e.g., cerebral edema), for the center, which kind of center (first, second, or third referral center) and if a pediatric diabetologist is always present at new patient admission. First and secondary referral centers have been considered all centers in which patients usually receive primary and secondary care from professionals such as a primary care physician (general practitioner or family physician), or medical specialists (pediatric diabetologists), who take care of less than 100 patients with type 1 diabetes. Tertiary referral centers are hospitals or university hospitals that provide tertiary care for more than 100 patients with type 1 diabetes in pediatric age.

In the final analysis the following data have been taken into consideration: number of patients 0–18 years of age with diabetes onset in the observation period, number of patients under 6 years of age, number of patients with DKA (either as total number or as patients under 6 years of age), number of patients with severe DKA (either as total number or as patients under 6 years of age), number of patients who presented cerebral edema, number of centers following any DKA protocol and which one, DKA management details (kind of fluid administered in the first and second hour, at which infusion rate, type of fluid used starting from the third hour, when insulin has been started and at which dosage, and usage of bicarbonate and in which circumstances), centers details like first/second referral centers or third referral centers, local epidemiology registry (yes or no), and presence of pediatric emergency department (yes or no).

DKA has been defined according to ISPAD biochemical criteria [6] (e.g., blood glucose > 11 mmol/L [≈200 mg/dL]; venous pH < 7.3 or bicarbonate < 15 mmol/L; ketonemia and ketonuria). The severity of DKA has been categorized by the degree of acidosis: (1) mild: venous pH < 7.3 or bicarbonate < 15 mmol/L; (2) moderate: pH < 7.2, bicarbonate < 10 mmol/L; (3) severe: pH < 7.1, bicarbonate < 5 mmol/L [6].

Data has been analyzed together for the calendar years 2012 and 2013. To evaluate possible statistical differences between groups, chi-square test has been used and a statistical significance has been determined at *p* < 0.05.

## 3. Results

Among the 77 centers belonging to the ISPED, 68 (87%) of them answered to our survey and returned complete data records of their newly diagnosed diabetes patients. Thirty-four centers are located in Northern Italy, 11 centers in Central Italy, and 23 centers in Southern Italy.

Among the 68 responders centers there are 100% of the tertiary referral centers of the ISPED so that the coverage of new diabetes cases in children 0–18 years of age has been pretty complete for the observational period (calendar years 2012 and 2013).

The 68 centers had a whole number of 14.493 children, adolescents, and young adults (under age 18) with type 1 diabetes. The tertiary referral centers were thirty-nine (each of whom take care for at least 100 patients or more), while primary/secondary referral centers were 29. Most of the 14.493 patients are followed up at the tertiary referral centers: 12.960/14.493 (89.4%).

The demographic characteristics of the participating centers are shown in Table 1. Most of them, especially tertiary referral ones (*p* < 0.001), have local registries, and only a minority (all tertiary referral ones) show a pediatric emergency unit. Looking at the person in charge for the DKA treatment, only a few (all tertiary referral centers) have dedicated personnel (pediatric diabetologist), while most leave DKA management to the general pediatrician, sometimes with the telephone help from a pediatric diabetologist.

In the years of the survey (2012/2013), a total of 2453 children 0–18 years of age have been diagnosed with type 1 diabetes in the 68 responder centers, and their records have been evaluated and reviewed for the present study. Unfortunately because of the retrospective nature of the survey, most of the data have been presented by each center in pooled format so that possible interesting analysis, like the distribution of age and gender of patients with DKA at onset, or the monthly incidence and local differences, has not been presented.

DKA has been observed in 945/2453 patients (38.5%), with severe DKA in 10.3% (Table 2). Evaluating DKA occurrence and tertiary versus primary/secondary referral centers, no difference has been observed either for total DKA (*p* = 0.562) or severe DKA (*p* = 0.893) (Table 2).

Evaluating DKA occurrence in preschool children (6 years of age or younger), total DKA has been observed in 445/618 patients (72%) (*p* < 0.001 versus patients >6 years of age) and severe DKA in 103/618 patients (16.6%) (*p* < 0.05 versus patients >6 years of age) (Table 2). These significant differences between all patients and preschoolers have been confirmed also when patients have been evaluated according to referral centers (Table 2).

Cerebral edema as a consequence of DKA management has been observed only in 5/945 patients (0.53%), all occurring in third referral centers.

All 68 responder centers declared to manage DKA according to written protocols: 46 centers (68%) use IDF/ISPAD guidelines [6], 15 centers used a protocol based on Lestradet indications [12], or slightly modified with respect to the one utilized during the IMDIAB study, called GETREM [13], and 7 centres (10%) used local protocols not referring to any international recommendation.

Regarding DKA management, all 68 responder centers declared to use 0.9% saline during the first 2 hours, but at different infusion rates: 48 centers (71%) infused saline at 5–10 mL/kg/h, 11 (16%) at 10–20 mL/kg/h, and 3 (4%) at less than 5 mL/kg/h, and 6 (8%) calculated infusion rate according to square meter (usually 3 L/m^2^ [12]).

After the first 2 hours, all 68 centers agree that the amount of fluids to be infused never have to exceed 3 L/m^2^/day, even if there was no agreement on the calculation (considering or not the amount of fluids infused during the first 2 hours). Moreover, a great difference has been observed regarding the nature of fluid infused after the first 2 hours: saline either 0.9% or 0.45% in 51 centers (75%), 5%–10% glucose solution, irrespective of glycemic values, in 13 (19%), and different solutions in the remaining 4.

Hypotonic solutions were never used when blood glucose was >14–18 mmol/L (>250–300 mg/dL); however, when blood glucose fell below that value, nonhypotonic solutions (containing at least 77 mEq/L of sodium) were used in 30 centers (44.1%), whereas glucose 5–10% solutions with sodium concentration ranging from 0 to 34 mEq/L (hypotonic) were used in 38 (65.9%).

Potassium was supplemented in all 68 centers, but supplementation was performed at different rates: 20–40 mEq/L in 43 (63%), calculated as mEq/kg/h in the remaining 25 (37%).

Sodium was not supplemented in any of the participating centers. However, because of its importance for determining osmolality it was determined in all patients in all centers.

Bicarbonates were never used in 22 centers (32%), while they were exceptionally used according to pH and clinical conditions in 46 (68%).

Regular insulin was infused using an automated syringe in 53 centers (78%), while it was injected directly in the fluid solution in 15 (22%). Insulin was infused starting from second or third hour in all 68 centers, mostly according to DKA severity. Insulin infusion rate was 0.05–0.1 U/kg/h in 49 centers (72%), while the rest used lower rates (0.02–0.07 U/kg/h).

Serum beta-hydroxybutyrate to rate DKA severity and to evaluate DKA management follow-up was measured in 43/68 centers (63%).

## 4. Discussion

The present survey provides a comprehensive depiction of DKA epidemiology and management in 68/77 centers that reviewed records of all newly diagnosed children 0–18 years old with type 1 diabetes in Italy during the calendar years 2012/2013.

Although the present data do not come from official diabetes registries and do not cover all the Italian country (11 centers did not send their data), we believe to give an enough satisfactory situation of DKA at diabetes onset in children younger than 19 years in Italy. This is because of the high number of patients represented by centers involved (*n* = 14.493 patients) and because all tertiary referral centers in Italy participated in the survey, accounting for quite 90% of patients involved in the study. Likewise, the centers that did not contribute to the survey are primary referral centers with a few patients each. Finally, the Italian Statistic Institute (ISTAT) esteems that the number of patients younger than 18 years of age in Italy is around 15.000–20.000.

Few years ago, Henriksen et al. [14] published a similar survey, led in adult patients with type 1 diabetes in Denmark. They achieved comparable country coverage (88% versus our 87%) and observed wide DKA treatment differences among centers, as we did, giving still higher reliability to our results.

Indeed, an interesting finding of the present survey is that, despite international recommendations, most of the Italian centers managing DKA in children with type 1 diabetes onset do not have a pediatric intensive care unit in their hospital, do not measure serum beta-hydroxybutyrate, and do not infuse insulin with an automated syringe. On the other hand, children with type 1 diabetes onset are managed by pediatric diabetologists in most of the cases, directly in 14%, or via phone consultation in 66%. Even with the lack of national regulation to claim the need for regional centers to manage all children with type 1 diabetes, this might be considered the result of the effective policy of our Society in actively promoting knowledge and expertise about type 1 diabetes management.

Another interesting finding of the present survey is the still too high number of children who had DKA at type 1 diabetes onset, with incidence (38.5%) similar to that reported in previous surveys led in Italy [10, 11, 15, 16], on smaller sample size and dating back to like 10–20 years ago. One possible reason might be that the Italian Health System (Sistema Sanitario Nazionale, SSN) is organized with family pediatricians who take care of children 0–14 years of age free of charge. Not everyone may be aware of the occurrence of type 1 diabetes, especially if they do not have patients suffering from this disease. Prevention campaign [4] succeeds in lowering DKA occurrence somewhere (Emilia Romagna region) but not everywhere, and we need an extra effort to gain similar results all over Italy; otherwise diabetes diagnosis can be delayed or missed at all. In this respect, family pediatricians can play a pivotal role in conducting effective DKA prevention campaigns able to reduce the incidence of DKA at type 1 diabetes onset, now unchanged for over twenty years.

Actually, the DKA incidence observed in the present survey is higher than that observed in many Northern European countries [1], and, recently, in the US SEARCH study [9], ranging around 30%. It should be remembered that both Northern European countries and the US show a higher type 1 diabetes incidence rate than Italy. In Italy the incidence rate is 12.26/100.000/year at the age 0–14 years, except in Sardinia that shows an incidence rate about 40/100.000/year [17]. DKA incidence is lower in higher type 1 diabetes incidence countries [1], and this could explain the still high Italian DKA incidence (38.5% versus 30%). Indeed, inferring Sardinian data from general ones we found a DKA incidence of 29%, very similar to that observed in the SEARCH study [9].

Italian DKA incidence is similar to that reported in a recent Austrian population-based study [18]. The authors describe a DKA incidence of 37.2% for all diabetes onsets in the period 1989–2011. It is noteworthy that the incidence reported is not different, as in our national data, before and after a prevention campaign similar to the Parma campaign [4]. In the study by Vanelli et al. DKA incidence decreased from 78% to only 12%, highlighting the fact that similar prevention campaign might work better when applied in a small area (Emilia Romagna region) instead of a national area (e.g., Italy and Austria).

No difference regarding DKA incidence as a whole has been observed between tertiary referral and primary/secondary referral centers. However, considering DKA in preschoolers [9, 18, 19], third referral centers showed a significantly higher incidence than primary/secondary referral ones. The explanation for this is probably the fact that in some area DKA episodes at diabetes onset (especially the more severe ones) in the preschoolers are referred from little hospitals to tertiary referral centers, without the possibility for the latter to lead a real prevention campaign or an early diagnosis of type 1 diabetes. This underlines the need of education and prevention programs, specifically addressed to this particularly vulnerable age group.

A great difference among centers has been observed also regarding the DKA management guidelines used. All agree to use 0.9% saline during the first 90–120 min; however, no agreement exists regarding the infusion rate, that differed largely according to the different protocols used. Similarly, a great difference exists regarding the type of fluids used after the first 2 hours.

While a general agreement occurs about the total amount of fluids to infuse (e.g., not to exceed 3 L/m^2^/day), differences have been observed in the methods to calculate it. This is probably due to the increasing evidence [20] that too many fluids are a risk factor for cerebral edema and possibly due to the fact that all pediatricians are more familiar with management of dehydration from various causes in children. The survey from Denmark in adult patients with type 1 diabetes [14] reported in the first 8 hours extensive differences in infused volumes, ranging from 3750 mL/day to 7700 mL/day, greater than the ones observed in the present survey.

There were no important disagreements in potassium and sodium infusion, possibly because there is a general agreement on their use in the existing guidelines.

Even though 68% of responder centers referred to the IDF/ISPAD guidelines [6], other protocols are still used, especially the “old” Lestradet protocol [12] consisting of an infusion of 10% glucose isotonic solution after 2-hour rehydration period. This may be explained by the historical importance of Lestradet protocol both in the ISPAD and in ISPED teaching schools and the fact that DKA treatment evidences are not of grade A level, highlighting the need for specifically designed randomized controlled trial, as the ongoing therapeutic trial recently published [21]. However, it is remarkable that when blood glucose starts dropping below 14–18 mmol/L (250–300 mg/dL), 56% of centers seem to not pay attention to using hypotonic solutions, as recently suggested by White and Dickson [22] who primarily focus on glucose concentration.

If all centers infuse human regular insulin, no agreement ensues regarding insulin infusion rate: most of centers infuse insulin at a rate of 0.05–0.1 U/kg/h, while 28% infuse insulin at a lower rate. Low dose is not inferior to standard dose with respect to rate of blood glucose decrease and resolution of acidosis, as showed previously [23] and in very recent randomized and controlled trial [24].

Cerebral edema, the most life-threatening complication of DKA, was reported in only few cases, with frequency (0.5%) similar to that described in a much larger case series by White and Dickson [22]. These data possibly suggest that, despite the nonhomogeneous attitude of Italian pediatric diabetologists to follow international guidelines, the protocols used rarely included bicarbonate infusion and the use of hypotonic solutions, factors associated with an increased risk of cerebral edema.

However, as well reviewed by Wolfsdorf in one of the last issues of Pediatric Diabetes, controversy persists concerning the appropriate starting dose of insulin for the treatment of DKA and the optimal fluid treatment regimen for pediatric DKA, also at an international level [25].

The present survey shows some limitations, the most significant being its retrospective design. Moreover, the establishment of a central registry will be of outmost importance for the validity and double check on the reliability of data collected. Additionally, total days of hospitalization, outcome of patients, and more demographic and epidemiological data need to be considered in future surveys on DKA in pediatric patients with type 1 diabetes.

In conclusion, the present survey describes an updated picture of what happens in Italy about incidence and management of DKA in children with type 1 diabetes. An awkwardly high DKA incidence at diagnosis has been observed, especially in preschool children, irrespective of specific prevention campaign carried out during the last years. Even if consistency in fundamental key points of DKA treatment has been perceived, some differences exist about treatment protocols, highlighting the need for their more effective sharing among centers [26].

## Figures and Tables

**Table 1:** Demographic characteristic of the 68 participating centers, according to being primary/secondary or tertiary referral centers.

	All (*n* = 68)	Primary/secondary referral centers (*n* = 29)	Tertiary referral centers (*n* = 39)	*p*
Local registries (*n*/%)	43 (63.2)	17 (58.6)	26 (66.6%)	<0.001
Pediatric emergency unit (*n*/%)	28 (41)	0 (0)	28 (71.8)	<0.0001
DKA treatment led by pediatric diabetologist (*n*/%)	10 (14)	0 (0)	10 (25.6)	<0.0001
DKA treatment led by general pediatrician with pediatric diabetologist on the phone (*n*/%)	45 (66)	16 (56.2)	29 (74.4)	<0.05
DKA treatment led by general pediatrician (*n*/%)	13 (19)	13 (19)	0 (0)	<0.0001

**Table 2:** Diabetic ketoacidosis incidence in the whole pediatric population (0–18 years) in the calendar years 2012 and 2013 in Italy.

	All (*n* = 68)	Primary/secondary referral centers (*n* = 29)	Tertiary referral centers (*n* = 39)	*p*
Total T1D patients (*n*/%)	14493 (100)	1533 (10.5)	12960 (89.5)	<0.0001
Patients with T1D onset (*n*/%)	2453 (100)	320 (13)	2133 (87)	<0.0001
Patients with DKA at T1D onset (*n*/%)	945 (38.5)	114 (35.6)	831 (39.0)	0.562
Patients with severe DKA at T1D onset (*n*/%)	253 (10.3)	35 (10.6)	218 (10.2)	0.893
Preschool patients with T1D onset (*n*/%)	618 (100)	542 (87.7)	76 (12.3)	<0.0001
Preschool patients with DKA at T1D onset (*n*/%)	445 (72)^*∗*^	414 (76.4)^*∗*^	31 (40.8)	<0.001
Patients with severe DKA at T1D onset (*n*/%)	103 (16.6)^*∗∗*^	94 (22.7)^*∗∗*^	13 (17.1)^*∗∗*^	<0.05

DKA = diabetic ketoacidosis; T1D = type 1 diabetes.

^*∗*^ *p* < 0.001 preschooler versus all; ^*∗∗*^ *p* < 0.05 preschooler versus all.
